# Efficacy of Switching to Levetiracetam After S-1-Induced Phenytoin Concentration Increase: A Case Report

**DOI:** 10.7759/cureus.82653

**Published:** 2025-04-20

**Authors:** Hayato Yokota, Haruka Igarashi, Yumiko Akamine, Shinichiro Atsumi, Akise Umakoshi, Masafumi Kikuchi

**Affiliations:** 1 Department of Pharmacy, Akita University Hospital, Akita, JPN; 2 Department of Gastroenterological Surgery, Akita University Graduate School of Medicine, Akita, JPN; 3 Department of Neuropsychiatry, Akita University Graduate School of Medicine, Akita, JPN

**Keywords:** adjuvant chemotherapy, antiepileptic drug, cyp2a6, interaction, levetiracetam, phenytoin, s-1, seizure

## Abstract

S-1, an oral anticancer drug, interacts with phenytoin (PHT) to increase PHT serum concentration. Although the PHT dosage is usually adjusted, few studies have examined the effects of switching from PHT to another antiepileptic drug. Here, we report the details of a case in which a patient with gastric cancer continued adjuvant chemotherapy after switching from PHT to levetiracetam (LEV) to prevent interactions with S-1. A man in his 60s with advanced gastric cancer received adjuvant chemotherapy with S-1 120 mg/day (cycles of two weeks of administration followed by one week of rest). He had experienced generalized tonic-clonic seizures 48 years prior and had been taking PHT (170 mg/day) and carbamazepine (250 mg/day). On day 22 of treatment, the PHT concentration increased from 3.72 to 11.76 µg/mL. On day 25, he developed dizziness and fell. Gradual PHT dose reduction and a switch to LEV improved his symptoms, and he remained seizure-free over nine treatment cycles. The findings of this case report suggest an approach for switching from PHT to another antiepileptic drug when PHT levels increase due to interactions with S-1. Switching to LEV may result in fewer interactions.

## Introduction

Phenytoin (PHT), widely used as an antiepileptic agent, requires dose adjustment through monitoring of blood concentrations because of its narrow therapeutic range [1]. Epilepsy treatment protocols begin with monotherapy, but refractory seizures often necessitate the addition of other antiepileptic drugs (AEDs). Multiple AEDs are metabolized by the cytochrome P450 (CYP) enzymes. PHT is metabolized primarily by CYP2C9 and CYP2C19, while carbamazepine (CBZ) undergoes metabolism via CYP3A4 [2]. Both drugs induce multiple CYP enzymes, including CYP1A2, CYP2C9, CYP2C19, and CYP3A4 [2]. Hence, patients with cancer taking these AEDs are at risk of drug interactions with oral anticancer agents. Previous reports have shown that 5-fluorouracil (5-FU) and its prodrugs - doxifluridine, capecitabine, and S-1 (a combination of tegafur, gimeracil, and oteracil potassium) - interact with PHT to increase PHT concentrations [3-7]. High PHT concentrations are toxic, causing symptoms such as nausea, vomiting, nystagmus, and motor impairment. Because of the nonlinear relationship between PHT dose and serum concentration [8], PHT therapy in combination with 5-FU requires frequent therapeutic drug monitoring and dose adjustments. No previous study has examined the clinical course after switching from PHT to another AED.

This report reviews the increase in PHT levels that occurred during adjuvant chemotherapy with S-1 in a patient with gastric cancer and focuses on the control of epilepsy through a switch from PHT to levetiracetam (LEV).

## Case presentation

The patient was a man in his 60s. During his childhood (48 years ago), he had developed generalized tonic-clonic seizures and received antiepileptic treatment with PHT. His electroencephalogram showed spike waves localized in the left temporal region. Occasionally, one type of epileptic seizure, automatisms, was observed. The patient had not had a seizure in 32 years. He was diagnosed with Stage Ⅲ advanced gastric cancer and underwent laparoscopic total gastrectomy with lymphadenectomy. After the operation, he was pathologically diagnosed with Stage IIIA gastric cancer and received adjuvant chemotherapy with S-1 plus docetaxel (DTX). Before treatment, he was taking PHT (170 mg/day) and CBZ (250 mg/day). PHT and CBZ serum concentrations were 3.72 μg/mL (dose-normalized concentration; C/D ratio: 0.02 μg /mL/mg) and 4.2 μg/mL (C/D ratio: 0.02 μg/mL/mg), respectively. Laboratory test values for liver and kidney function remained stable, with aspartate transaminase, alanine transaminase, total bilirubin, and creatinine levels within normal ranges and an albumin level of 3.5 g/dL.

**Figure 1:**
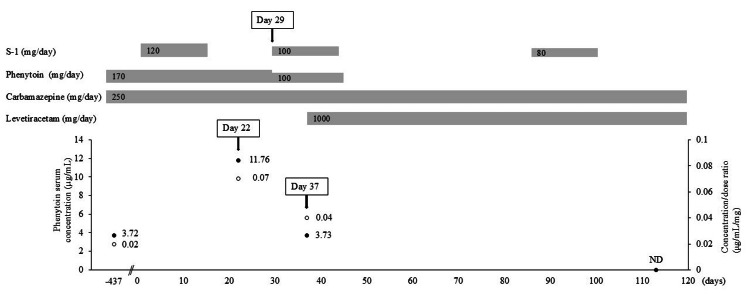
Clinical course of the patient The doses of S-1, phenytoin, carbamazepine, and levetiracetam; phenytoin serum concentration (black circles); and concentration/dose ratio (white circles) are shown. ND: not detected.

He was treated with S-1 (120 mg/day) during the first course on days 1 to 14 of a three-week cycle. From the second course, he was scheduled to receive an intravenous infusion of DTX (40 mg/m2 body surface area) on day 1 of each cycle and S-1 on days 1 to 14 of a three-week cycle. At the start of the second course (day 22), the pharmacist confirmed the patient’s medication history and raised concerns about a potential interaction between S-1 and PHT. The patient had begun experiencing numbness in his toes. The pharmacist recommended measuring PHT concentrations, which were subsequently tested. Meanwhile, laboratory tests revealed leukopenia (white blood cells (WBC): 2,000/µL, Grade 2), neutropenia (neutrophils (NEUT): 900/µL, Grade 3), and thrombocytopenia (platelets (PLT): 53,000/µL, Grade 2), which resulted in the postponement of the second course of S-1 plus DTX (Table 1). On day 25, the patient tripped while walking, requiring a visit to the emergency department. Four days later (day 29), the PHT concentration and C/D ratio of the blood sample collected on day 22 were confirmed to have increased more than threefold to 11.76 μg/mL and 0.07 μg/mL/mg, respectively (Figure 1). After psychiatric consultation, the PHT dose was reduced to 100 mg/day. The second cycle of chemotherapy resumed with S-1 at a reduced dose of 100 mg/day, while DTX remained postponed. On day 37 (cycle 2, day 9), the PHT concentration had decreased to 3.73 μg/mL (C/D ratio: 0.04 μg/mL/mg), and LEV 1,000 mg/day was prescribed. The patient remained seizure-free, and PHT was completely discontinued by day 44 (cycle 2, day 16). On day 50 (cycle 2, day 22), laboratory tests showed decreased numbers of WBC (2,700/µL) and NEUT (1,400/µL); furthermore, Grade 3 thrombocytopenia (PLT: 35,000/µL), leading to the postponement of the third cycle of S-1 plus DTX. On day 85 (cycle 3, day 1), S-1 was resumed at a reduced dose of 80 mg/day. On day 113, CBZ levels had increased to 5.7 μg/mL (C/D ratio: 0.02 μg/mL/mg), while PHT was undetectable. After the fourth cycle, S-1 treatment at 80 mg/day was continued on a schedule of two weeks of administration followed by a one- or two-week drug withdrawal period, depending on WBC and platelet counts. CBZ (250 mg/day) and LEV (1,000 mg/day) were continued, and no seizures occurred throughout the nine treatment cycles.

**Table 1:** Laboratory test values before and after treatment

Day	1	22	29	50	57	71	85	113
White blood cells (/μL)	4,400	2,000	5,200	2,700	4,200	4,500	3,700	2,400
Neutrophils (/μL)		900	3,500	1,400	3,100	3,000	2,700	
Platelets (×10^3^/μL)	196	53	183	35	131	128	146	62
Hemoglobin (g/dL)	11.3	10.9	11.1	10.2	10.2	10.4	10.1	10.0
Albumin (g/dL)	3.5	3.8	3.9	3.9	3.7	3.6	3.4	3.6
Creatinine (mg/dL)	0.76	0.78	0.75	0.80	0.74	0.80	0.80	0.73

## Discussion

In this patient with gastric cancer, we observed increased PHT concentrations following S-1 administration, with a 3.2-fold increase in the PHT C/D ratio. Few studies have assessed changes in the PHT C/D ratio [9]. Moreover, we switched the treatment course from PHT to LEV to prevent interactions with S-1. Several case reports have shown that PHT has been switched to LEV or lacosamide to avoid interactions with 5-FU [5-7]; however, the subsequent clinical course has not been examined in detail. Frequent monitoring of PHT concentrations was difficult because the patient was an outpatient. Furthermore, since myelosuppression is a dose-limiting toxicity of 5-FU [10], the intensity of the interaction between PHT and 5-FU becomes more complex when the 5-FU dosage or treatment schedule is altered. Previous studies have found that PHT treatment increases the risk of bone fractures [11], and this patient had previously fallen twice and visited the emergency department. Therefore, we considered discontinuing PHT and either stopping antiepileptic medication entirely or switching to another AED. Discontinuation of AEDs is considered in children after a period of seizure remission of more than two years [12]. In contrast, the optimal timing for discontinuation in adults remains unclear. Lamberink et al. reported that the risk of seizures decreased with every additional seizure-free year [13]. We considered that the risk of seizure recurrence was low, given that the patient had been seizure-free for over 30 years and maintained stable PHT and CBZ concentrations. A randomized controlled trial involving 1,013 adults with epilepsy who had been in remission for over two years demonstrated that the use of more than one AED and a history of tonic-clonic seizures were risk factors for recurrence in patients who discontinued their medication [14]. However, many seizure-free patients may be able to maintain their seizure-free status, even after switching to another medication [15]. PHT was gradually tapered and discontinued, and we switched the patient to LEV, another AED, because of his history of tonic-clonic seizures. LEV may be preferred over PHT from the perspective of drug-drug interactions. 5-FU decreased the protein expression of both CYP2C11 and CYP3A enzymes in rats [16]. In addition, Gunes et al. reported reduced CYP2C9 activity in patients with colorectal cancer treated with 5-FU [17]. Therefore, we hypothesized that S-1 administration caused delayed PHT metabolism, increasing PHT concentrations. In contrast, LEV undergoes partial metabolism through hydrolysis, independent of CYP enzymes [18], and is widely used for both generalized and partial epilepsy [19]. Therefore, LEV may serve as an alternative option in cases of drug-drug interactions, such as those observed with the coadministration of S-1 and PHT.

In this case, the serum concentration of PHT increased approximately threefold on the eighth day (day 22) after discontinuing S-1. This suggests an interaction with 5-FU generated from the metabolism of tegafur, as similar reports have shown that the effects of S-1 on the blood concentration of PHT persisted until day 14 after S-1 discontinuation [20]. On the 11th day after discontinuing S-1 (day 25), the patient experienced dizziness, stumbled, and fell. These symptoms were resolved after the PHT concentration decreased. The patient had no history of cardiovascular disease. Previous reports have shown that symptoms of PHT toxicity emerged more than one month after the start of combined treatment with fluoropyrimidine drugs such as 5-FU [4]. The therapeutic range for PHT is 10-20 μg/mL [1]. However, the PHT concentration required to complete seizure control may vary depending on seizure type, the severity of the underlying disorder, and genetic abnormalities [21]. In a single-center retrospective study, a total of 159 patients (73 females, 86 males) receiving epilepsy treatment with both PHT and CBZ had an average plasma PHT concentration of 6.24 μg/mL, while that in patients receiving PHT alone was 6.42 μg/mL [22]. Therefore, PHT levels may be maintained in real-world clinical practice at lower concentrations. However, several case reports showed that even when PHT is maintained below its therapeutic range, concomitant 5-FU administration may increase PHT levels to 20 μg/mL or higher [23,24]. Therefore, even if PHT levels are low before S-1 administration, clinicians should be prepared for a rapid increase in PHT concentration with concomitant administration of S-1.

During the treatment course, S-1 monotherapy caused Grade 3 thrombocytopenia and neutropenia, resulting in an inability to administer DTX. In the domestic phase III trial (JACCRO GC-07 trial) of adjuvant therapy, Yoshida and coworkers reported that the incidence of Grade 3 or higher myelosuppression with S-1 monotherapy was 2% for leukopenia, 16.1% for neutropenia, and 0.3% for thrombocytopenia [25]. This intriguing finding could be attributed to the enzyme-inducing effect of CBZ on S-1 metabolism. Tegafur, a component of S-1, is metabolized primarily to 5-FU via 5'-hydroxylation mediated by CYP2A6 [26], and CBZ induces CYP2A6 [27]. Meanwhile, LEV did not affect CYP2A6 metabolic activity in human liver microsomes and showed no CYP-inducing effects in rat hepatocytes [28]. Our patient continued treatment for nine cycles with a reduced dose of S-1 and an extended drug discontinuation period.

## Conclusions

Because PHT has nonlinear pharmacokinetics, clinicians find it challenging to predict the degree of drug interaction with concomitant S-1. This case report proposes an approach for switching from PHT to another AED in patients with epilepsy in remission when PHT levels increase due to interactions with S-1. LEV may be an appropriate alternative, with fewer interactions.
